# Band-limited implicit neural representations for diffusion-weighted imaging denoising

**DOI:** 10.1088/1361-6560/ae2a9e

**Published:** 2026-01-08

**Authors:** Yunxiang Li, Yan Dai, Yen-Peng Liao, Jie Deng, You Zhang

**Affiliations:** Department of Radiation Oncology, University of Texas Southwestern Medical Center, Dallas, TX 75390, United States of America

**Keywords:** diffusion-weighted imaging, denoising, implicit neural representation, self-supervised learning

## Abstract

*Purpose.* Diffusion-weighted imaging (DWI) has significant value in disease diagnosis and treatment response monitoring, but its inherent low signal-to-noise ratio (SNR) severely affects image quality and quantification accuracy. Existing denoising techniques often blur important tissue boundary information when suppressing noise. *Methods.* This study proposes a band-limited implicit neural representation (BL-INR) framework for DWI denoising. The method introduces BL positional encoding based on the frequency response characteristics of the sinc function to restrict INR models from learning high-frequency noise while maintaining strong signal representation capabilities. Furthermore, multi-*b*-value DWI and structural MRI from the same patient are integrated as anatomical priors, exploiting the correlation between true signals and the statistical independence of noise to achieve effective denoising. *Main Results.* In clinical DWI data evaluation across four anatomical regions (brain, head and neck, abdomen, and pelvis), BL-INR’s visualization results were superior to existing methods. Under extremely low SNR conditions (SNR = 1) in simulated noise experiments, BL-INR achieved a peak SNR of 35.44 and structural similarity index of 0.933, significantly outperforming other methods. Phantom denoising results showed that BL-INR achieved an average apparent diffusion coefficient value error of only $4.57 \times 10^{-5}$ mm^2^ s^−1^, the smallest among all methods. *Significance.* BL-INR provides a novel approach for DWI denoising by limiting the frequency of INR input positional encoding. Its self-supervised learning characteristics require no paired training data and allow convenient clinical application. The method enables the derivation of accurate diffusion parameters, providing a reliable foundation for DWI-based quantitative analysis with significant clinical application value.

## 1. Introduction

Diffusion-weighted imaging (DWI) is a magnetic resonance imaging technique based on water molecule diffusion motion, playing a vital role in neurological disease diagnosis, cancer detection, and treatment response evaluation (Katti *et al* 2011, Koh and Le Bihan 2003). DWI provides important information about tissue microstructure and pathological changes by measuring the degree of water molecule diffusion restriction in tissues. However, DWI images are typically affected by low signal-to-noise ratios (SNRs), primarily due to their special imaging mechanism—diffusion-sensitive gradient application leads to signal attenuation (Jones and Cercignani 2010, Tournier *et al* 2011). Low SNR not only degrades image quality but also leads to inaccurate estimation of diffusion parameters (such as apparent diffusion coefficient (ADC)), thereby affecting clinical diagnosis and treatment decisions (Aja-Fernández *et al* 2009, Veraart *et al* 2016).

To improve DWI image quality, researchers have proposed various denoising methods. Traditional image processing methods such as Gaussian filtering and median filtering can suppress noise to some extent but often blur image details simultaneously, especially under low SNR conditions (Buades *et al* 2005, Zhang *et al* 2017). More advanced approaches such as non-local means (NLMs) (Buades *et al* 2005), block-matching, and 4D filtering (BM4D) (Maggioni *et al* 2013), and variational methods (such as Rudin–Osher–Fatemi total variation) (Rudin *et al* 1992) can better preserve edge information but still show limitations in complex texture regions. Deep learning methods have achieved significant progress in image denoising (Zhang *et al* 2017, Koonjoo *et al* 2021), but most methods require large amounts of paired training data (noisy images and corresponding clean images), which are difficult to obtain in the DWI field. To address this issue, researchers have proposed a series of self-supervised learning methods, such as Noise2Self (N2S) (Batson and Royer 2019), Noise2Void (N2V) (Krull *et al* 2019), and Patch2Self (P2S) (Fadnavis *et al* 2020), which can train using only noisy images without clean reference images. These methods achieve effective denoising by exploiting the statistical independence of noise and inherent redundancy of true signals.

**Figure 1.**
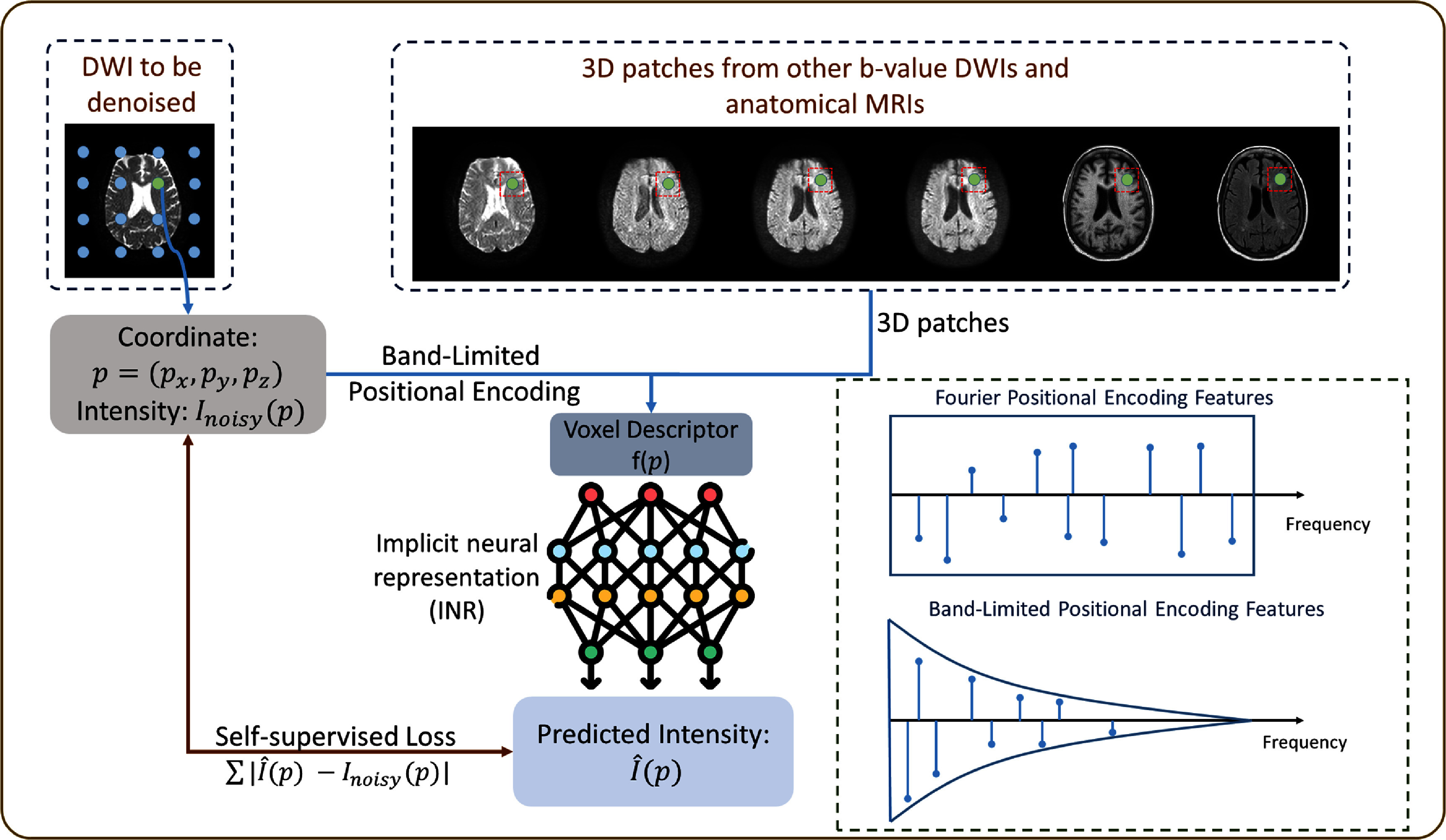
Overview of the band-limited implicit neural representation (BL-INR) framework. The framework combines two types of input information: (1) band-limited positional encoding that applies frequency constraints to spatial coordinates through sinc functions; (2) functional/anatomical prior information mapping with multi-*b*-value DWI and structural MRI guidance. This information is processed through a lightweight multilayer perceptron (MLP) network to predict denoised DWI images.

However, these self-supervised methods primarily focus on local feature extraction and pixel-level prediction, with limited capability to model global image structure and continuous signal characteristics. This limitation becomes particularly evident in DWI, where anatomical structures demonstrate inherent spatial continuity, and diffusion parameters typically exhibit smooth variations within homogeneous tissue regions. To address this fundamental challenge, recent advances in neural signal representation offer promising alternatives. INR, as an emerging signal representation method, has drawn widespread attention in computer vision in recent years (Sitzmann *et al* 2020, Mildenhall *et al* 2022, Li *et al* 2025a, 2025b). INR uses neural networks to directly learn continuous mapping functions from spatial coordinates to signal values, possessing inherent advantages in preserving signal continuity. The commonly used Fourier positional encoding transforms spatial coordinates into high-dimensional sinusoidal features to help neural networks learn high-frequency signals, equipping INR with detail learning capabilities (Rahimi and Recht 2007, Tancik *et al* 2020). Such high-frequency signals, however, may include noise as well (Jacot *et al* 2018), defeating the purpose of denoising. However, without coordinate encoding, the expressive power of INR models would be severely limited because of the low-frequency spectral bias of MLPs, which are the primary backbone of INRs. Therefore, effectively maintaining INR learning capabilities while restricting high-frequency noise learning remains a key challenge in DWI denoising applications. Consequently, this study proposes a self-supervised learning framework based on BL-INR for DWI denoising (as shown in figure 1). DWI denoising is crucial for clinical practice, as it directly impacts the accuracy of extracted quantitative diffusion parameters that are used for disease diagnosis, treatment planning, and therapeutic response monitoring. Improved image quality enables more reliable detection of subtle pathological changes and enhances confidence in clinical decision-making. The main contributions of this study can be summarized as follows:

(1) introduction of BL positional encoding that effectively suppresses high-frequency noise while preserving important anatomical structure information by limiting the frequency range of positional encoding;
(2) incorporation of functional/anatomical structure priors provided by multi-*b*-value DWIs and anatomical MRIs (such as T1, T2) to enhance the structural accuracy during denoising (Fischl *et al* 2002, Despotovic *et al* 2015);
(3) adoption of a self-supervised learning strategy that directly learns denoising mapping from noisy images without requiring paired clean images as training data (VanBerlo *et al* 2024);
(4) comprehensive evaluation on simulated and clinical DWI data from multiple anatomical sites (brain, head and neck (HN), abdomen, and pelvis), demonstrating the effectiveness and generalizability of the proposed method.

## 2. Methods

### 2.1. Model overview

Let $\Omega \subset \mathbb{R}^3$ represent the image domain, considering a noisy DWI volume acquired at a target *b*-value (e.g. *b* = 0 or any specific *b*-value of interest). For each voxel $\mathbf{p} = (p_x, p_y, p_z) \in \Omega$, we seek to estimate the underlying noise-free signal that preserves structural contrast while suppressing noise artifacts. The fundamental assumptions of our method are: (1) MRI signals are spatially continuous, while noise has a low correlation with surrounding voxels; (2) MRI noise is random and weakly correlated between different acquisitions, while true image structures are shared across images of the same patient. Based on the two assumptions, we employ band-limited (BL) positional encoding to capture signal continuity features, helping to distinguish signals from noise. Meanwhile, since true image structures have a high correlation between different images of the same patient, we can use other *b*-value DWIs (excluding the DWI at the queried target *b*-value) and anatomical MRIs as functional/anatomical prior information to map the target image for denoising. In contrast, due to its low correlation among different images, noise will be much more difficult for the model to learn during this mapping process, thus effectively achieving denoising.

The proposed BL-INR method uses a lightweight MLP to regress the target DWI signal $\hat{I}(\mathbf{p})$ from each voxel’s descriptor $\mathbf{f}(\mathbf{p})$. The voxel descriptor $\mathbf{f}(\mathbf{p})$ includes BL positional encoding and functional/anatomical priors from 3D patches of contextual MRIs. The network architecture consists of four fully connected layers, each with a hidden dimension of 1024 units, with the final layer outputting the predicted noise-free intensity for the target voxel.

**BL positional encoding:** we propose a novel BL positional encoding to represent spatial information, which achieves intrinsic frequency limitation based on the frequency response characteristics of the sinc function. The sinc function is chosen as it is the time-domain response of an ideal low-pass filter, providing a strict zero response at the cutoff frequency and effectively preventing overfitting to high-frequency noise. For voxel $\mathbf{p} = (p_x, p_y, p_z)$, we first normalize its coordinates to the $[-1,1]$ range. Let the frequency set be $\{\omega_k\}_{k = 1}^{K_{\omega}}$, where frequencies *ω*_*k*_ are equally spaced within the interval $[1, \omega_{\max}]$. The maximum frequency $\omega_{\max}$ is empirically determined through ablation studies (detailed in section 3.6) to balance spatial detail preservation and noise suppression. To ensure that the normalized sinc function $\text{sinc}(x\sigma) = \sin(\pi x\sigma)/(\pi x\sigma)$ equals exactly 0 at the maximum frequency, we set $\sigma = 1/\omega_{\max}$, utilizing the zero-point characteristic of the normalized sinc function at integer values to achieve the ideal frequency cutoff. For each dimension $i \in \{1,2,3\}$ and frequency *ω*_*k*_, the sine and cosine components of BL positional encoding are calculated as:

\begin{align*} \phi_{i,k}^{\sin} &amp; = \sin\left(\omega_k p_i\right) \cdot \text{sinc}\left(\omega_k \sigma\right),\end{align*}

\begin{align*} \phi_{i,k}^{\cos} &amp; = \cos\left(\omega_k p_i\right) \cdot \text{sinc}\left(\omega_k \sigma\right).\end{align*}

Through the multiplicative factor of the sinc function, high-frequency components automatically attenuate with increasing frequency, achieving a natural low-pass filtering effect. The complete BL positional encoding vector $\boldsymbol{\phi}(\mathbf{p})$ is formed by concatenating all sine and cosine components across dimensions and frequencies.

**Functional/anatomical prior information mapping:** our multi-modal prior fusion strategy is based on a key observation: while multi-*b*-value DWIs and structural MRIs from the same patient exhibit different contrast mechanisms and signal characteristics, they share the same underlying anatomical structure. Specifically, our fusion process comprises three key steps: first, for each voxel position in the target *b*-value DWI to be denoised, we extract local neighborhood information from the corresponding positions in all other *b*-value DWIs and available structural MRIs (T1, T2, etc). Second, these multi-source local patches are flattened and concatenated to form a high-dimensional feature vector that encodes rich functional and anatomical contextual information. Finally, this fused feature vector, together with the BL positional encoding, is fed into the neural network to guide the denoising reconstruction of the target voxel. The core advantage of this design lies in the fact that genuine anatomical signals are highly correlated across different modalities, while noise components are more statistically independent. Therefore, the network can learn to extract cross-modal consistent signal patterns while suppressing uncorrelated noise. Let $\mathcal{B} = \{b_1, b_2, \ldots, b_K\}$ be the set of all available *b*-values in the acquisition protocol excluding the target *b*-value. For each $b_k \in \mathcal{B}$, we extract a $3 \times 3 \times 3$ patch $\mathbf{P}_{b_k}(\mathbf{p}) \in \mathbb{R}^{27}$ centered at voxel **p**. Signal intensities at different *b*-values are interconnected through the same tissue microstructural properties, but their respective noise components are statistically more independent. Therefore, by aggregating local information from multiple *b*-values, we can effectively enhance the SNR of the latent representation while providing the network with rich context about local diffusion properties. When co-registered anatomical scans (such as T1-weighted, T2-weighted, or proton density images) are available, we can further extract corresponding $3 \times 3 \times 3$ patches $\mathbf{P}_{s_j}(\mathbf{p})$ from each anatomical modality *j*. Anatomical images share the same anatomical foundation as DWI but possess higher contrast and clearer tissue boundaries, providing additional anatomical constraints for the network. This incorporation of anatomical information helps maintain gray-white matter interfaces, sulci, and other anatomical landmarks that may be blurred in highly noisy DWI data.

In summary, the complete voxel descriptor is formed by concatenating all available information:

\begin{align*} \mathbf{f}\left(\mathbf{p}\right) = \left[\underbrace{\boldsymbol{\phi}\left(\mathbf{p}\right)}_{\text{band-limited PE}}, \underbrace{\mathbf{P}_{b_1}\left(\mathbf{p}\right), \ldots, \mathbf{P}_{b_K}\left(\mathbf{p}\right)}_{\text{multi-}\textit{b}\ \text{local}}, \underbrace{\mathbf{P}_{s_1}\left(\mathbf{p}\right), \ldots, \mathbf{P}_{s_M}\left(\mathbf{p}\right)}_{\text{anatomical local}}\right],\end{align*}

where $\boldsymbol{\phi}(\mathbf{p})$ is the BL positional encoding vector, *K* is the number of available multi-*b*-value DWIs (excluding the target *b*-value), and *M* is the number of anatomical MRIs.

### 2.2. Self-supervised learning objective

Given the concatenated coordinate vector and contextual patches, the network *g*_*θ*_ predicts the true (noise-free) intensity of the target voxel:

\begin{align*} \hat{I}\left(\mathbf{p}\right) = g_\theta\left(\mathbf{f}\left(\mathbf{p}\right)\right),\end{align*}

where *θ* represents the network parameters. During training, the model observes the noisy target image itself as supervision. The learning objective is formulated as a mean squared error loss:

\begin{align*} \mathcal{L} = \frac{1}{|\Omega|} \sum_{\mathbf{p} \in \Omega} \left(I_{\text{noisy}}\left(\mathbf{p}\right) - g_\theta\left(\mathbf{f}\left(\mathbf{p}\right)\right)\right)^2,\end{align*}

where $I_{\text{noisy}}(\mathbf{p})$ is the observed noisy intensity at voxel **p**. The key insight enabling this self-supervised approach is based on the statistical properties of signal and noise in MRI data. Specifically, we leverage the fact that: (1) image signals exhibit spatial smoothness and are highly correlated between different *b*-values and anatomical contrasts of the same patient, while (2) noise components are more statistically independent between different acquisitions and have limited spatial correlation. Mathematically, let $S(\mathbf{p})$ and $N(\mathbf{p})$ represent the true signal and noise at voxel **p**, respectively. The observed intensity is $I_{\text{noisy}}(\mathbf{p}) = S(\mathbf{p}) + N(\mathbf{p})$. Since noise $N(\mathbf{p})$ is weakly correlated between different *b*-values and anatomical modalities (Aja-Fernández and Tristán-Vega 2013), the network learns to extract the consistent signal component $S(\mathbf{p})$ that explains the correlated patterns in the multi-source input features $\mathbf{f}(\mathbf{p})$, while the weakly correlated noise component cannot be consistently predicted from the functional/anatomical priors. This leads to effective denoising at test time while preserving consistent features represented across input modalities.

Notably, our method adopts a patient-specific training strategy, training a dedicated BL-INR model for each target *b*-value DWI of each patient. Specifically, for patients with multiple *b*-values (such as $b = 0, 50, 400, 800$, etc), we train an independent model for each target *b*-value requiring denoising, with other *b*-value DWIs and anatomical MRIs serving as functional/anatomical prior inputs. Furthermore, our self-supervised learning framework requires only noisy images as training data, without any clean reference images (ground truth). It makes BL-INR particularly appealing for DWI denoising tasks, as it is difficult to obtain truly noise-free DWIs as supervision signals in clinical practice.

## 3. Experimental results

### 3.1. Experimental setup

#### 3.1.1. Clinical dataset

We conducted comprehensive evaluations using multi-site clinical DWI data to validate the effectiveness and generalizability of BL-INR. Clinical DWI data were collected from different anatomical sites, with each case defined as a complete DWI examination session including all *b*-value acquisitions and associated anatomical MRI sequences acquired during a single patient visit:

• **Brain DWI:** 47 cases of brain DWI data
• **Head and neck (HN) DWI:** 7 cases of HN DWI data
• **Abdominal DWI:** 20 cases of abdominal DWI data
• **Pelvic DWI:** 5 cases of pelvic DWI data

While the dataset includes multiple examination sessions from some patients acquired on different dates, each case represents a complete examination session from a single visit. Our self-supervised training strategy ensures complete independence between training processes for each case, eliminating any training-testing data leakage concerns. Since model training requires only the data from each individual case (self-supervision), the training dataset of each case is also the test dataset, without requiring additional training/test set partitioning. In our study, we used clinical DWI acquisition protocols for MR-guided radiotherapy on MR-LINACs. This protocol acquires diffusion-weighted images along only three orthogonal directions (*X, Y, Z*) at each *b*-value and generates trace-weighted images through averaging. The clinical workflow saves and exports only the trace-weighted images for diagnosis and quantitative analysis. Therefore, our BL-INR method is directly applied to denoise trace-weighted images.

For each case, since both DWIs and structural MRIs were acquired from the same scanning session pre-denoising image registration was unnecessary. We used DWI as the reference imaging space and resampled the structural MRIs (T1- or T2-weighted) to the DWI voxel grid through trilinear interpolation, thereby achieving resolution/coordinate matching. This approach, based on the same-session data, effectively avoids registration errors that could arise from cross-session scans. Scanning parameters varied slightly across cases, with *b*-values spanning: 0, 5, 50, 100, 200, 400, 500, 600, 800, 1000, 1500, 2000, 2500, and 3000 s mm^−2^. The numbers and types of anatomical MRI sequences also varied, encompassing T1-weighted, T2-weighted, and T2-FLAIR sequences. This diversity in data characteristics enables robust validation of our method’s generalizability across different scanning protocols and parameter configurations.

**Simulated dataset:** of the 47 brain DWI cases, 33 originated from MR-LINAC systems, while 14 were acquired using MR Simulator systems. MR Simulator systems, designed specifically for high-precision treatment planning, feature superior magnetic field homogeneity, advanced gradient systems, and optimized radiofrequency coils compared to MR-LINAC systems, as the latter prioritize on-board imaging during radiation delivery. This fundamental difference in system design results in substantially lower noise levels and higher image quality in MR Simulator acquisitions. Therefore, we leveraged B0 images from all MR Simulator cases as the foundation for simulated data generation. The B0 images were selected due to their highest signal intensity and minimal noise contamination, providing near noise-free reference standards. We artificially introduced Rician-distributed noise (the established noise model for DWI) to these B0 images, generating noisy versions with SNR levels of 1, 3, and 5. This simulation approach, grounded in authentic clinical data, provides a controlled noise environment for quantitative denoising performance evaluation with access to noise-free references.

Using the simulated brain dataset, we quantitatively studied the optimal $\omega_{\max}$ parameter that offers a balance between denoising and structure preservation. Additionally, to evaluate the generalizability of the $\omega_{\max}$ parameter across different anatomical regions, we conducted similar simulation experiments on the aforementioned abdomen, head-and-neck, and pelvis data. Since the original B0 images in these regions inherently have higher noise levels and lack truly noise-free reference images, we adopted an alternative strategy: we first used the BL-INR method to denoise the B0 images from these regions, obtaining relatively clean images as reference standards. While these denoised data are not suitable for direct performance comparison with other methods (due to potential bias), they serve as a reasonable alternative for evaluating the optimal $\omega_{\max}$ value across different anatomical regions.

**Implementation details:** We trained the network with the Adam optimizer, starting from an initial learning rate of $4 \times 10^{-4}$ and decaying it using a cosine-annealing schedule. Training was performed for 20 epochs on an NVIDIA RTX 4090 GPU.

#### 3.1.2. Comparison methods

We selected representative traditional and deep learning-based methods as comparison baselines. Traditional methods included: Gaussian filtering (Gaussian), median filtering (Median), NLMs (Buades *et al* 2005), wavelet thresholding denoising (Wavelet) (Chang *et al* 2000), BM4D (Maggioni *et al* 2013), Perona–Malik diffusion (P–M) (Guo *et al* 2011), and Rudin–Osher–Fatemi total variation (ROF-TV)(Rudin *et al* 1992). Deep learning approaches comprised: N2S (Batson and Royer 2019), N2V (Krull *et al* 2019), P2S (Fadnavis *et al* 2020), multi-modal blind-spot network (MMBSN) (Zhang *et al* 2023), and zero-shot Noise2Noise (ZSN2N) (Mansour and Heckel 2023). Fair comparison was ensured by utilizing official implementations or widely recognized open-source versions, with parameters configured according to recommendations in the respective publications.

#### 3.1.3. Evaluation metrics

We implemented a multi-dimensional evaluation framework to assess denoising performance with tailored strategies for different data types.

**Simulated data evaluation:** for simulated data derived from MR Simulator systems, the availability of near noise-free reference images enables the evaluation of traditional full-reference image quality metrics: (1) peak SNR (PSNR), which quantifies overall denoising effectiveness through the logarithmic representation of mean squared error between denoised and reference images, with higher values indicating superior noise suppression; (2) structural similarity index (SSIM) (Wang *et al* 2004), which assesses image quality from a human visual perception perspective by simultaneously evaluating brightness, contrast, and structural information, providing comprehensive measurements of structural preservation during denoising, with values approaching one indicating optimal structural integrity; (3) learned perceptual image patch similarity (LPIPS) (Zhang *et al* 2018), which measures perceptual similarity based on deep neural network features, with lower values indicating better perceptual quality. Additionally, for simulated data evaluation, we applied Rician noise bias correction as a post-processing step. This correction involves measuring a background noise intensity and subsequently removing the noise floor using the widely adopted mathematical formulations (Gudbjartsson and Patz 1995), ensuring more accurate quantitative assessment of the denoising performance.

**Phantom data evaluation:** phantom data offers unique evaluation advantages through laboratory-determined ground-truth ADC values, enabling precise quantitative assessment of ADC maps computed from DWIs processed by different denoising methods. In this study, we also scanned a DWI physical phantom and measured the ADC signals to compare differet methods (see section 3.4). We calculated errors between ADC maps derived from denoised DWIs and true laboratory-determined ADC values using mean absolute error (MAE). It provides the most reliable standard for validating denoising method performance in preserving diffusion parameter accuracy, which is crucial for assessing the clinical applicability of denoising methods in quantitative DWI analysis.

**Clinical data evaluation:** for the original clinical DWI data lacking true noise-free references, traditional PSNR and SSIM metrics cannot be evaluated. We therefore employed reference-free evaluation methods based on signal statistical characteristics. Specifically, we selected regions of interest (ROIs) with relatively uniform signal in each case and measured pixel mean and standard deviation within these regions. According to fundamental denoising principles, effective denoising methods should substantially reduce signal standard deviation (indicating noise suppression) while preserving signal mean values (indicating tissue contrast preservation). This evaluation strategy eliminates dependence on ground truth while directly evaluating the performance of denoising methods in actual clinical environments. Additionally, we visualized ADC value changes before and after denoising to assess each method’s impact on diffusion parameter estimation. Note that for clinical DWI data, Rician noise correction was not applied as post-processing, since the MRI data exported from clinical scanners undergo vendor-specific processing pipelines that make it difficult to accurately measure the noise level required for proper Rician correction.

### 3.2. Simulated data results

**Figure 2.**
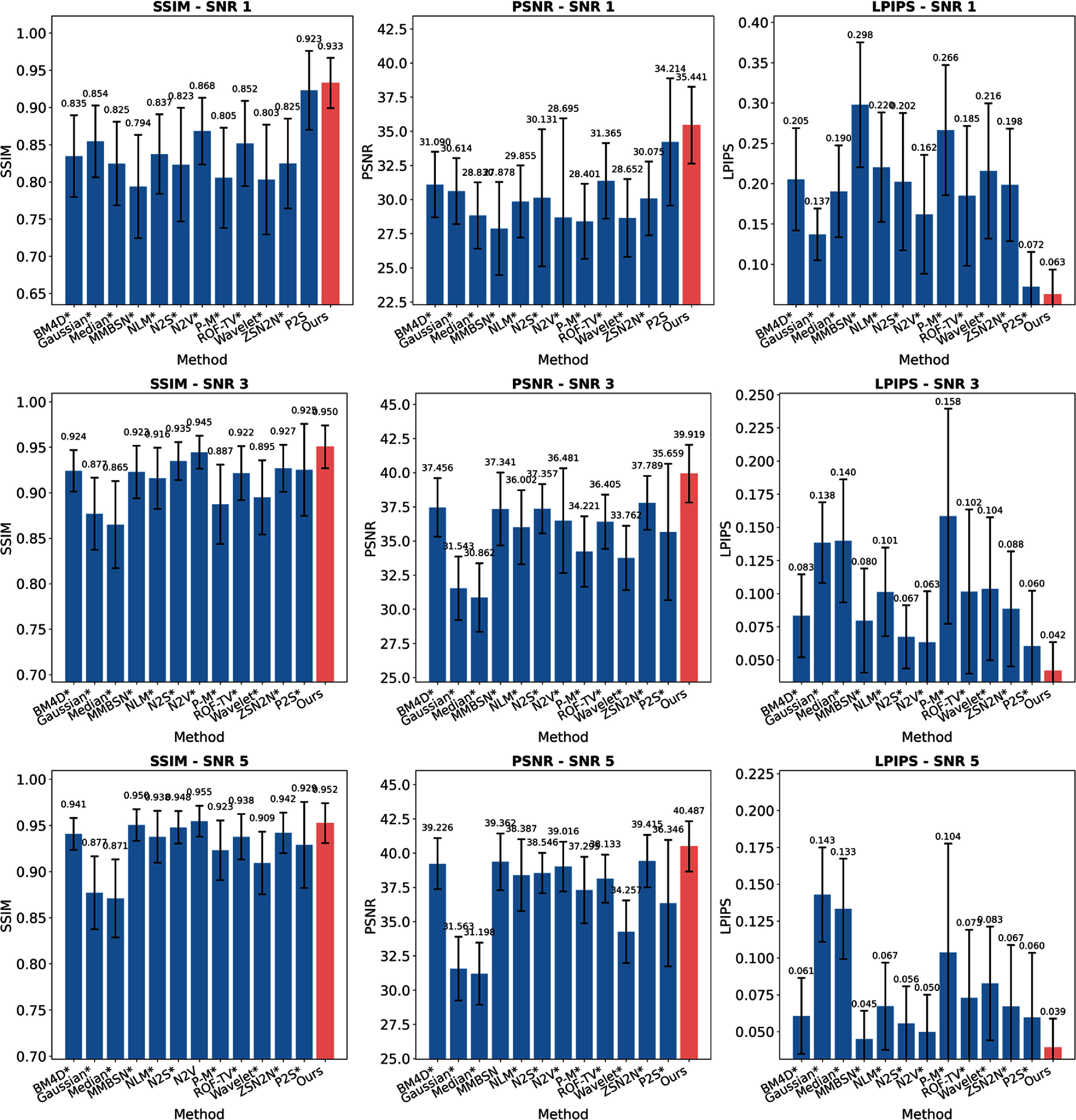
Quantitative evaluation results of various denoising methods under different SNR conditions (SNR = 1, 3, 5). The three rows correspond to SNR = 1, 3, 5 conditions, and the three columns show comparisons of SSIM, PSNR, and LPIPS metrics respectively. Error bars represent standard deviation. Asterisks (*) indicate statistically significant differences compared to BL-INR (*p* $ < $ 0.05, Wilcoxon signed-rank test).

**Figure 3.**
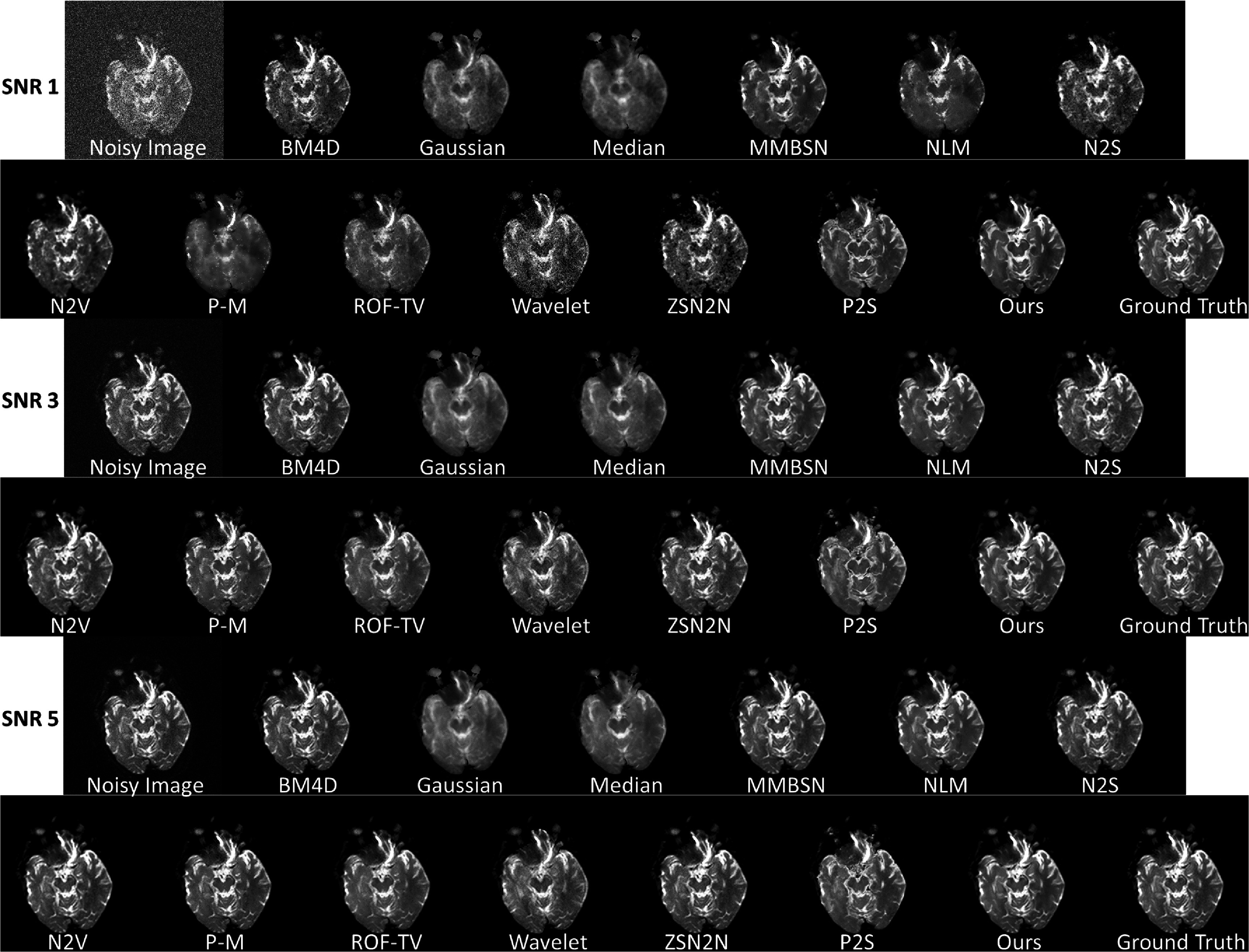
Visual comparison results of various denoising methods under different SNR conditions (SNR = 1, 3, 5).

Figures 2 and 3 comprehensively compare BL-INR against existing methods under simulated SNR conditions of 1, 3, and 5. The quantitative evaluation (figure 2) employs three complementary metrics: SSIM assesses structural fidelity, PSNR quantifies overall signal quality, and LPIPS measures perceptual similarity. Statistical significance was evaluated image-wise using the Wilcoxon signed-rank test. Under extremely low SNR conditions (SNR = 1), BL-INR achieves the best performance across all three metrics with SSIM of 0.933, PSNR of 35.44 dB, and LPIPS of 0.063, where PSNR outperforms the second-best P2S by 1.2 dB. As SNR increases to 3 and 5, all methods show performance improvements, but BL-INR maintains its leading advantage, reaching SSIM of 0.952, PSNR of 40.49 dB, and LPIPS of 0.039 at SNR = 5. The visual comparison (figure 3) reveals distinct denoising characteristics of different methods: traditional filters like Gaussian, wavelet, and median blur anatomical boundaries while reducing noise; BM4D and NLM preserve more structures but show visible noise artifacts under low SNR conditions; self-supervised methods (N2S, N2V) perform better overall but occasionally produce unnatural textures; recent deep learning methods MMBSN and ZSN2N show improvements over traditional approaches but still underperform BL-INR under extremely low SNR conditions. In contrast, BL-INR effectively suppresses noise across all SNR levels while faithfully preserving delicate structural details, including gray-white matter boundaries, cortical sulci, and gyri.

### 3.3. Clinical DWI experimental results

We comprehensively evaluated BL-INR’s performance across multiple anatomical sites, including the brain, HN, abdomen, and pelvis regions. Each anatomical site presents distinct imaging challenges and technical requirements, providing rigorous assessment of our method’s adaptability and clinical utility.

**Figure 4.**
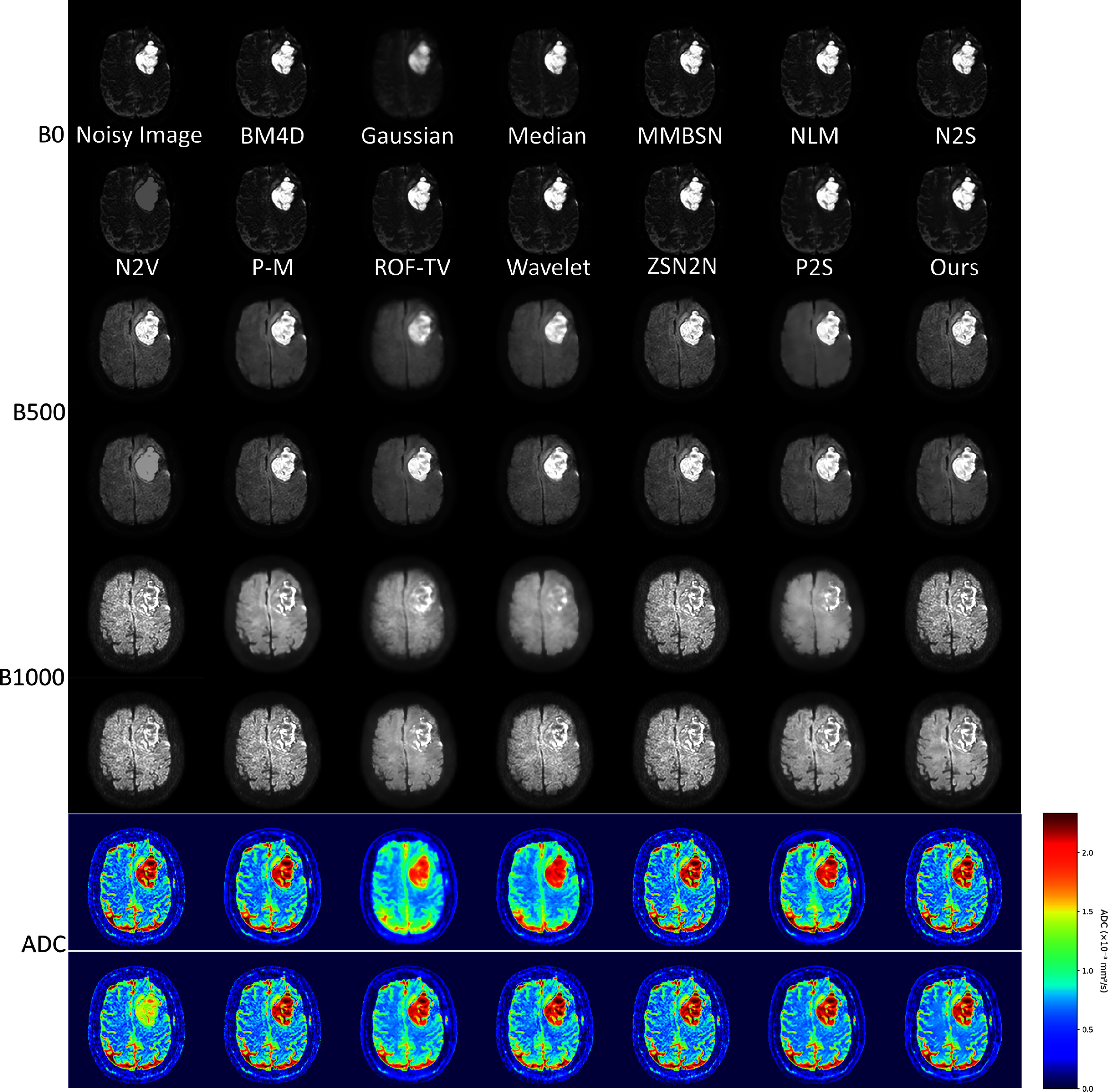
Comparison of brain DWI denoising results. BL-INR demonstrates superior performance in preserving structural details while effectively suppressing noise.

Brain DWI presents unique challenges due to complex structures and varying tissue contrasts. Figure 4 compares the performance across different denoising approaches. Traditional filtering approaches (Gaussian, wavelet, and median) achieve noise reduction but at the expense of critical structural features, blurring cortical patterns and gray-white matter interfaces that are essential for neurological assessment. Advanced denoising methods such as BM4D, NLM, P–M, and ROF-TV have shown improvements over traditional approaches like Gaussian and wavelet filtering. However, they still present certain limitations. For example, the results of P–M still exhibit noticeable residual noise, while ROF-TV tends to cause over-smoothing, as seen in the ADC results, leading to the loss of fine anatomical structures. Self-supervised approaches (N2S, N2V) demonstrate improved overall performance but occasionally generate texture artifacts at tissue interfaces. In contrast, BL-INR produces visually superior results, effectively eliminating noise while maintaining cortical morphology, tissue boundaries, and white matter tract integrity.

**Figure 5.**
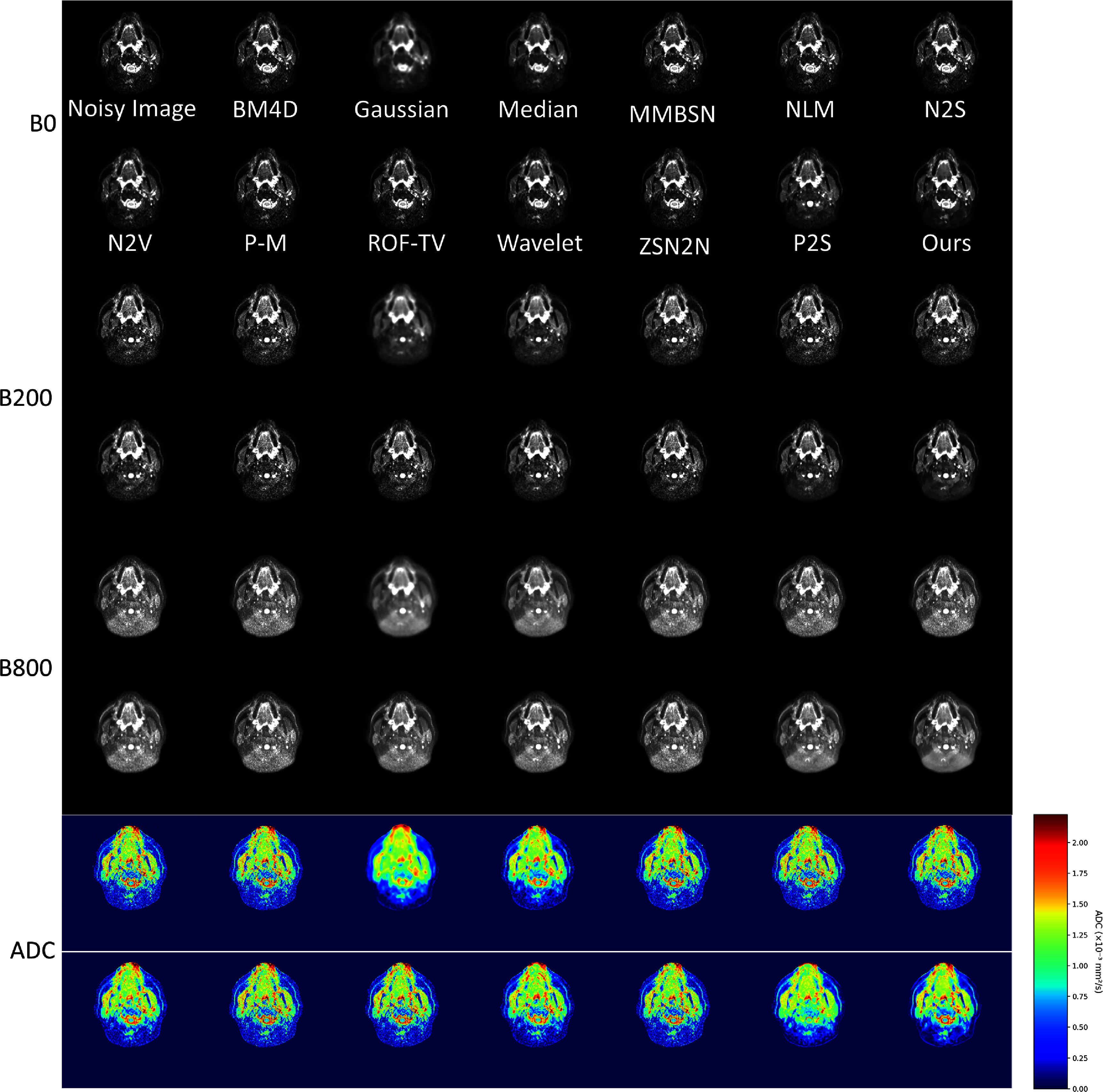
Comparison of head and neck DWI denoising results. The complex structures in head and neck regions pose unique challenges for denoising algorithms, and BL-INR maintains excellent performance.

**Figure 6:**
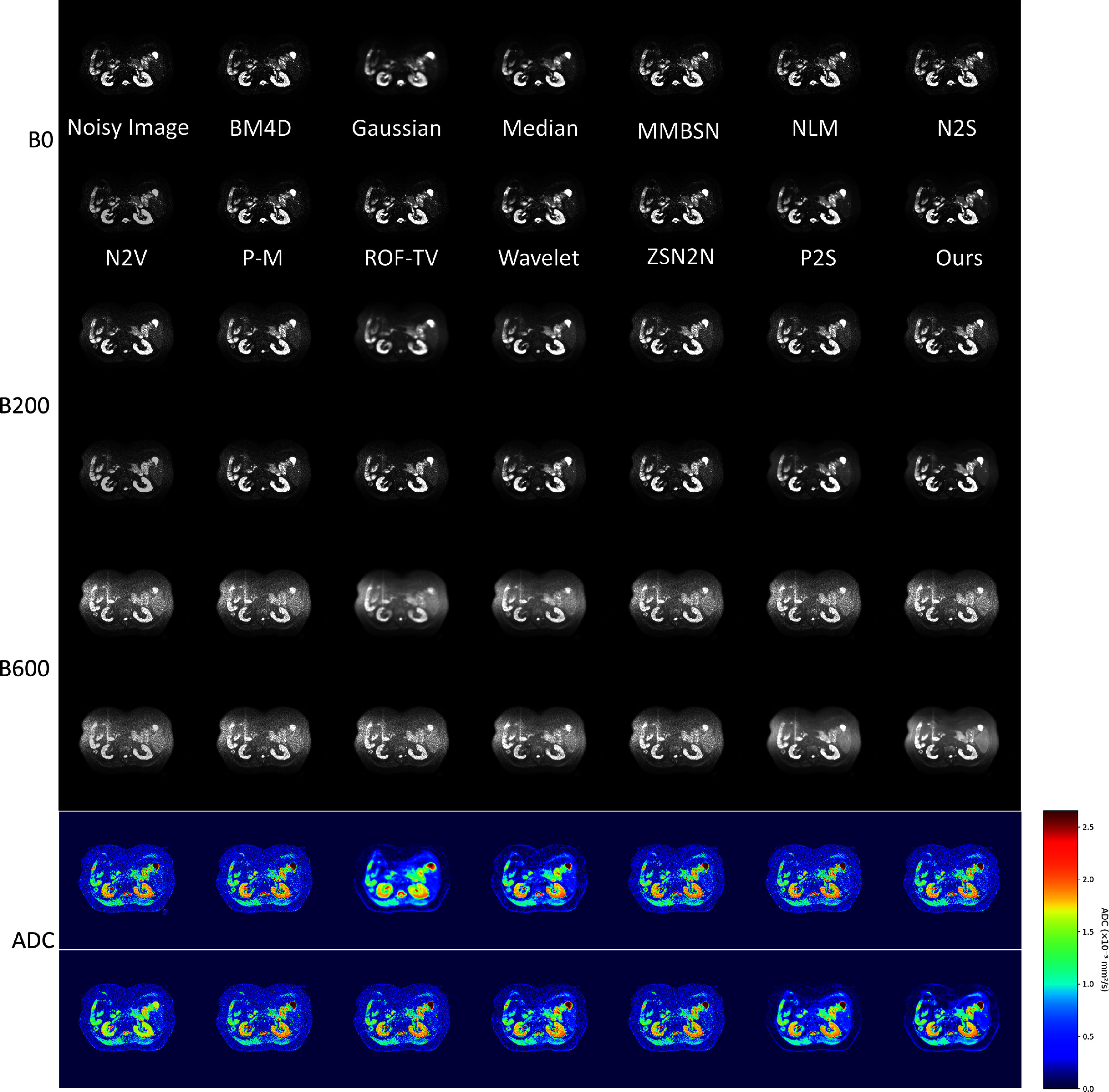
Comparison of abdominal DWI denoising results. Abdominal imaging faces challenges from respiratory motion and complex organ distribution, and BL-INR demonstrates robust denoising performance.

**Figure 7.**
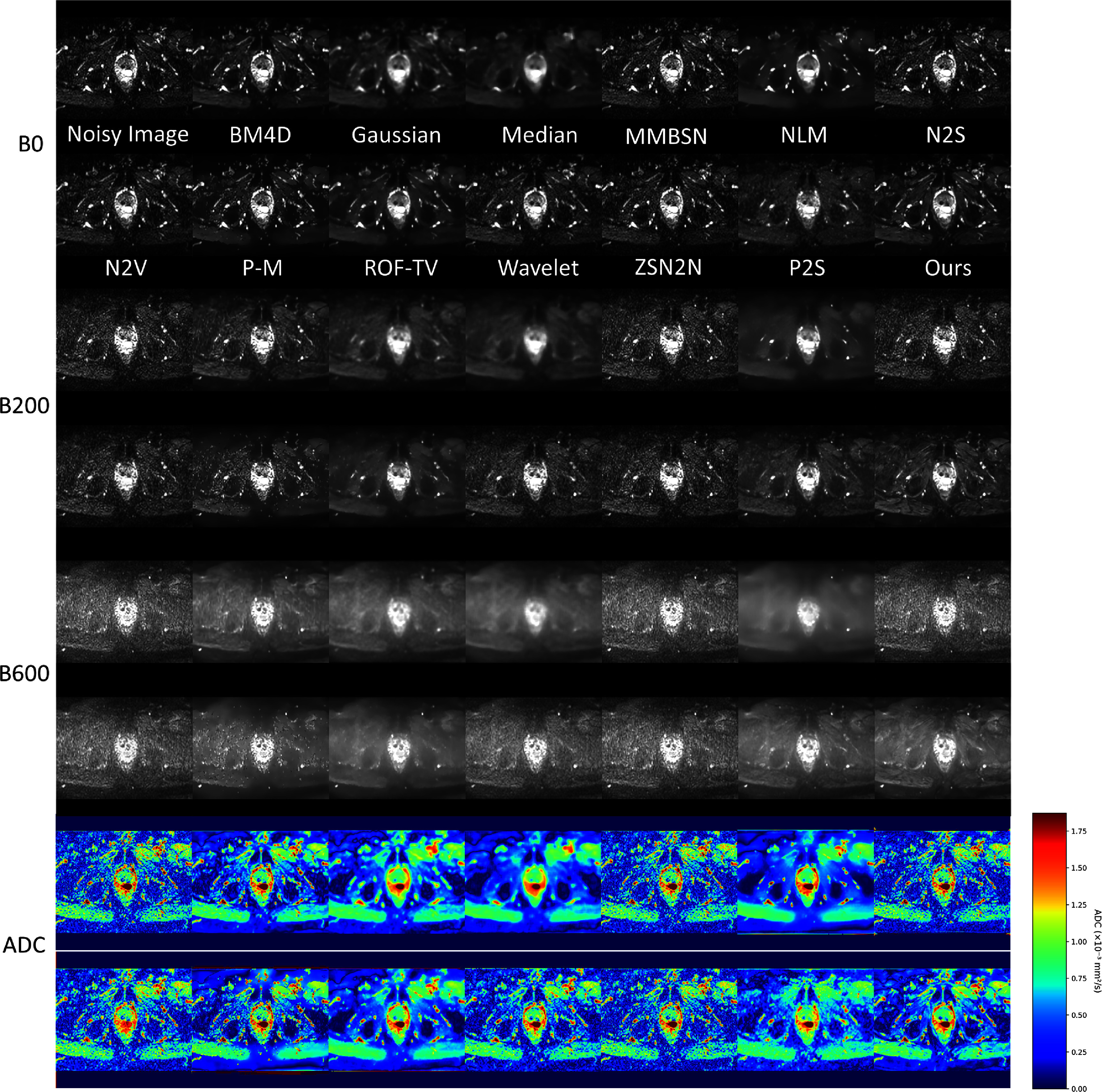
Comparison of pelvic DWI denoising results. In pelvic imaging with complex soft tissue structures, BL-INR effectively preserves tissue contrast while suppressing noise.

Beyond brain imaging, we systematically evaluated BL-INR across HN, abdominal, and pelvic regions (figures 5–7). HN imaging faces challenges from complex anatomical geometry and susceptibility artifacts. Despite these difficulties, BL-INR successfully preserves delicate structures, including salivary glands, lymph nodes, and muscle tissue boundaries, while achieving effective noise suppression. Abdominal DWI presents distinct challenges from respiratory motion, organ heterogeneity, and varying signal characteristics across different tissues. BL-INR demonstrates robust performance, maintaining sharp organ boundaries between the liver, kidney, pancreas, and surrounding structures. Similarly, pelvic imaging involves intricate soft tissue structures critical for urological and gynecological applications. BL-INR effectively maintains tissue contrast between the bladder, reproductive organs, and surrounding soft tissues. Additionally, we provide evaluation results of BL-INR for intravoxel incoherent motion model parameter fitting in the Supplementary material, demonstrating the method’s excellent performance in more complex diffusion models as well.

**Figure 8.**
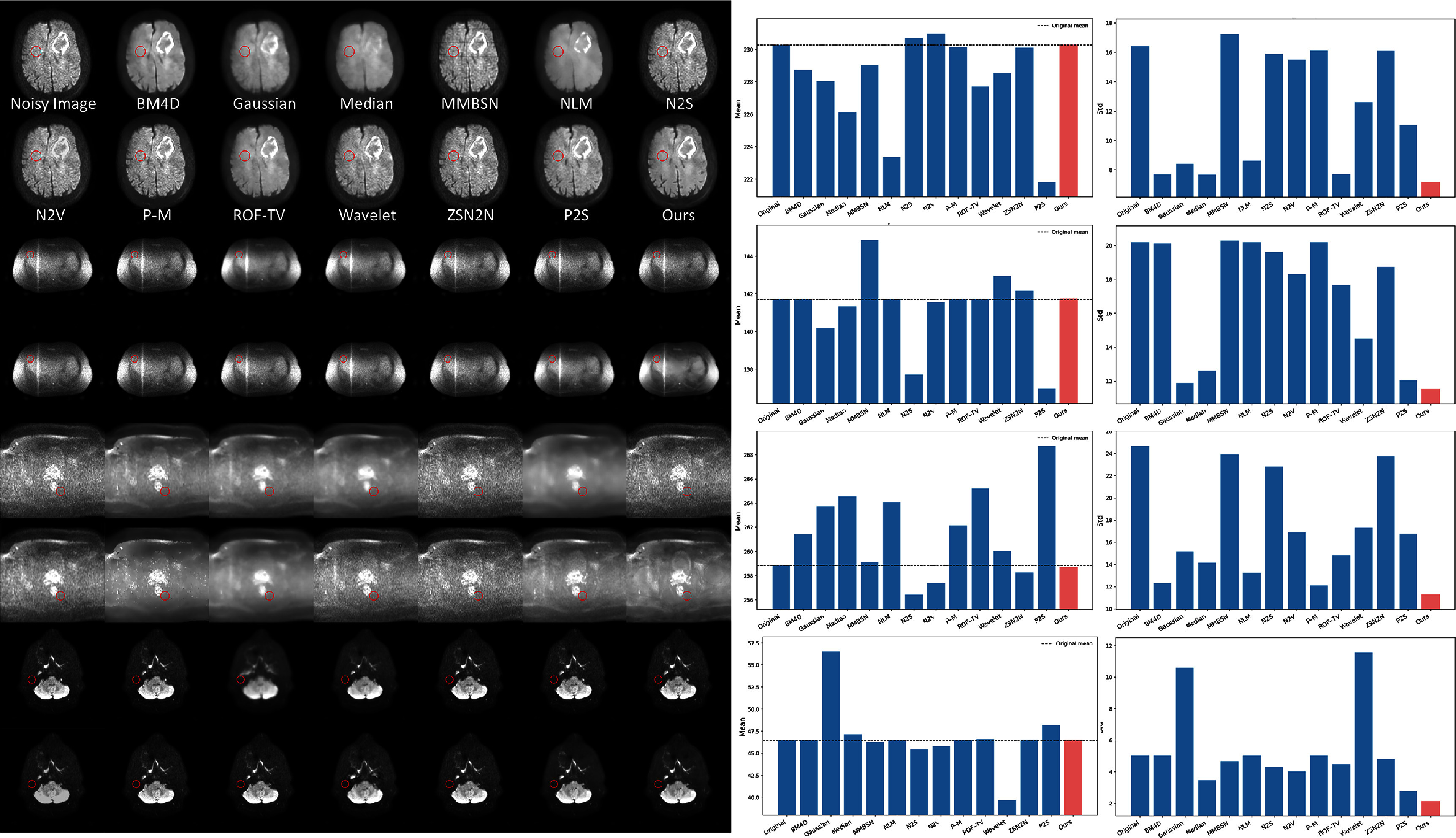
Quantitative statistical analysis of DWI denoising results across different anatomical regions. The figure shows the mean and standard deviation changes of signal intensity in selected regions of interest before and after denoising by different methods for brain, head-and-neck, abdomen, and pelvis. Each subplot demonstrates the noise suppression capability (standard deviation reduction) and signal fidelity (mean preservation) of each method.

Quantitative validation across all anatomical sites corroborates the visual observations. Figure 8 presents statistical analyses of signal characteristics before and after denoising across brain, head-and-neck, abdominal, and pelvic regions. The ROI statistical analysis across all four anatomical sites demonstrates that BL-INR consistently achieves the greatest standard deviation reduction compared to other denoising methods while preserving the mean signal intensity. Most notably, for pelvic data, our BL-INR model reduces the standard deviation to 40% of the original noisy images, representing the largest improvement among all anatomical regions. This consistent performance across diverse anatomical regions confirms BL-INR’s robustness.

### 3.4. Phantom experimental results

**Figure 9.**
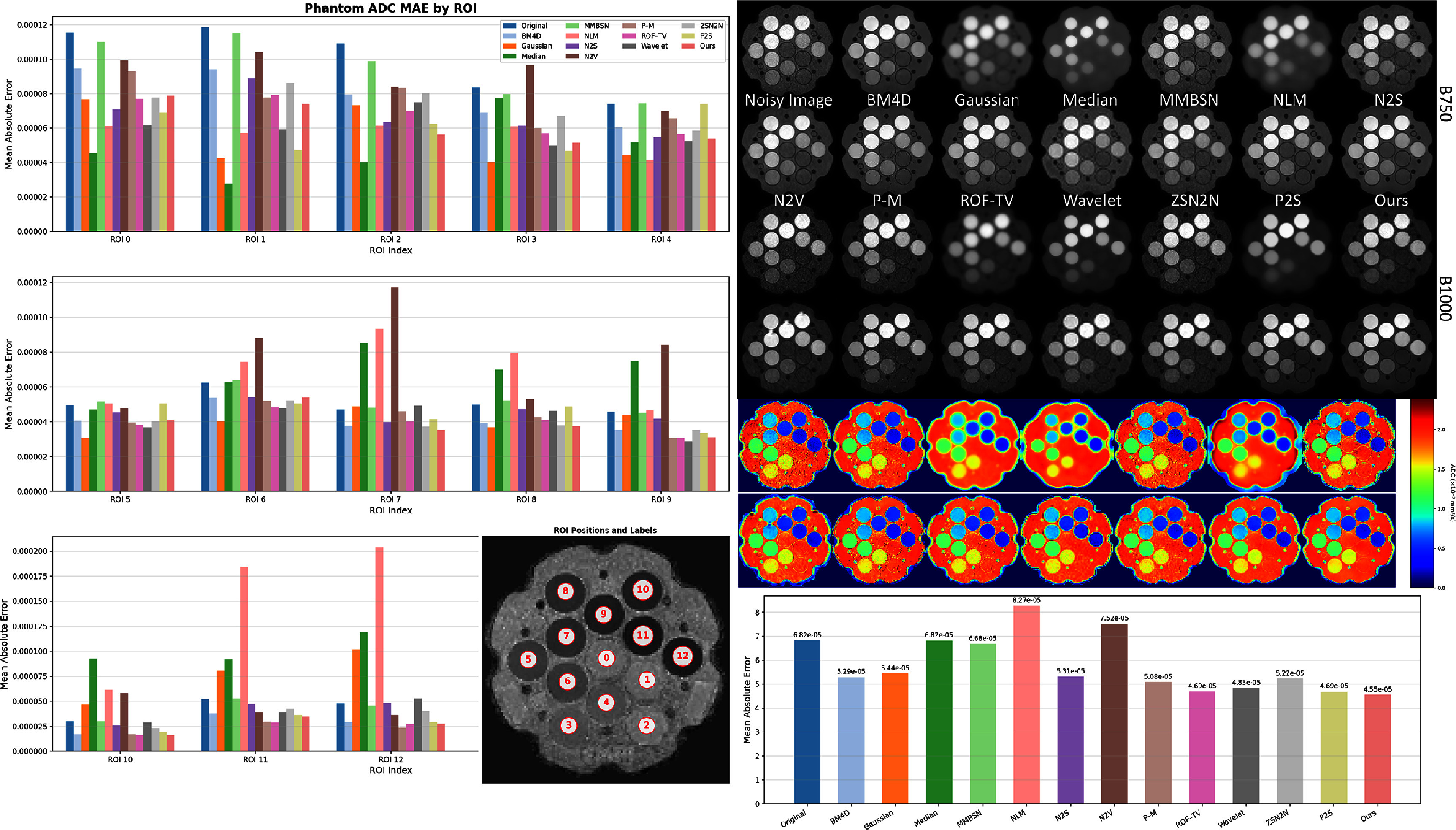
Diffusion phantom experimental results. The left panel presents detailed MAE analysis for each of the 13 individual tubes, revealing performance variations across different diffusion characteristics; the upper right shows visual comparisons of various denoising methods at B750 and B1000 DWIs along with corresponding ADC parameter maps; the lower right bar chart displays the average MAE across all 13 tubes. All MAE values are expressed in ADC standard units of mm^2^s^−1^.

We evaluated BL-INR’s performance in deriving diffusion parameters using a standard diffusion phantom (Keenan *et al* 2016). The phantom serves as a gold-standard reference tool, containing multiple test tubes with known ADC values that simulate human tissue diffusion characteristics (Tournier *et al* 2011). For all experiments, ADC maps were computed using least-squares fitting of the mono-exponential diffusion model across all available *b*-value DWIs from each case, following the standard equation $S(b) = S_0 \exp(-b \cdot \text{ADC})$, where *S*(*b*) represents the signal intensity at *b*, and *S*_0_ is the signal intensity at *b* = 0. Figure 9 demonstrates the performance evaluation of various denoising methods. The tube-by-tube MAE analysis on the left reveals how different methods handle varying diffusion characteristics. Traditional filtering approaches (Gaussian, wavelet, and median) show consistently high errors across most tubes due to boundary blurring and signal over-smoothing that compromise ADC calculation accuracy. While BM4D and NLM preserve boundaries better, they still exhibit significant parameter bias in certain tubes. Self-supervised deep learning methods (N2S, N2V, P2S) improve upon traditional methods, but texture artifacts at tube-background interfaces lead to non-uniform error distributions. The visual comparison in the upper right panel further confirms BL-INR’s advantage, as it maintains optimal geometric integrity and signal uniformity at both B750 and B1000 *b*-values.

ADC parameter maps provide validation of quantitative accuracy. Traditional methods exhibit spatial non-uniformity and parameter shifts, particularly at tube edges, where smoothing effects cause signal mixing and boundary blurring. While deep learning methods reduce these artifacts, they still show local parameter distortions. When comparing its ADC maps with the laboratory standard values, BL-INR achieves the lowest average MAE (as shown in the lower right bar chart), reducing the error by approximately 33% compared to the original noisy images, outperforming all other denoising methods. This improvement carries significant clinical implications, as ADC accuracy directly impacts reliability in disease detection, treatment monitoring, and prognosis assessment (Koh and Collins 2007, Padhani *et al* 2009).

### 3.5. Ablation study

**Figure 10.**
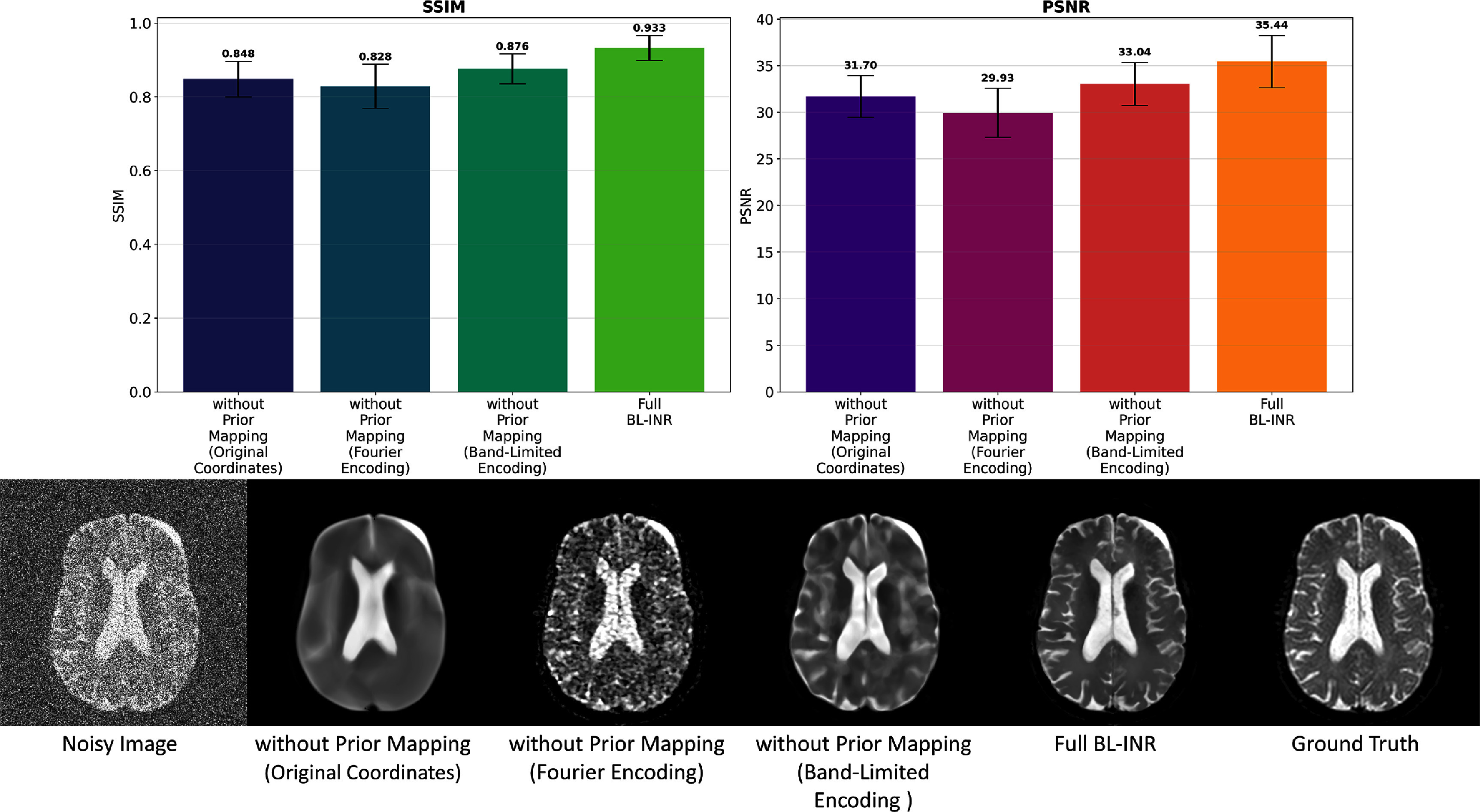
Ablation study results of the BL-INR framework. The upper part shows a quantitative comparison of different model variants on SSIM and PSNR metrics, and the lower part presents visual denoising effects of each variant, including noisy images, without prior mapping (original coordinates), without prior mapping (Fourier encoding), without prior mapping (band-limited encoding), the full BL-INR model, and ground-truth images. Results demonstrate that band-limited positional encoding and multi-modal prior information fusion are crucial for improving denoising performance.

We conducted ablation experiments to evaluate each component’s contribution in the BL-INR framework. To ensure controlled experimental conditions and enable quantitative evaluation with ground-truth references, we utilized the simulated dataset with SNR = 1 from our brain DWI collection, which represents the most challenging noise scenario and provides the most stringent test for component effectiveness. By progressively removing or replacing core elements in the framework, we can quantify each component’s impact on overall performance and gain a deep understanding of their synergistic effects. Figure 10 presents results of these ablation experiments. In ablation experiment design, we constructed four key model variants to systematically evaluate each component’s contribution: **without prior mapping (original coordinates)** variant that directly uses normalized spatial coordinates as network input without any frequency domain transformation, and excludes multi-modal DWI/MRI prior mapping; **without prior mapping (Fourier encoding)** variant that employs the standard Fourier positional encoding, and excludes multi-modal DWI/MRI prior mapping; **without prior mapping (BL encoding)** variant that uses only our proposed BL positional encoding strategy, and excludes multi-modal DWI/MRI prior mapping; finally, the **Full BL-INR** model integrates all design components, including BL positional encoding and multi-modal prior information fusion.

Quantitative evaluation results reveal progressive contributions of each component to model performance. In terms of SSIM, the variant without prior mapping (original coordinates) performs worst with a mean SSIM value of 0.848, demonstrating the fundamental importance of positional encoding mechanisms for the INR model’s expressive power. The low-frequency bias characteristics of raw coordinates make it difficult for networks to capture high-frequency details in images, resulting in over-smoothed reconstruction results that lose important anatomical structural features. The variant without mapping (Fourier encoding) shows a decrease compared to raw coordinates, with the mean SSIM dropping to 0.829. This result supports our core hypothesis: standard Fourier encoding, when processing noise-contaminated medical images, indiscriminately enhances all frequency components, including high-frequency noise, due to its full-spectrum characteristics, thus limiting further improvement of denoising effects. Our proposed BL positional encoding strategy demonstrates significant performance advantages, with the mean SSIM value improving to 0.876. It demonstrates the core value of frequency-selective representation: through carefully designed frequency cutoff mechanisms, BL encoding can effectively suppress high-frequency noise components while preserving mid-to-low frequency signals that contain important structural information. From PSNR, BL encoding achieves a 3.1 dB improvement compared to Fourier encoding (33.04 dB vs 29.93 dB). The full BL-INR model, by integrating multi-modal prior information, achieves the best performance across all evaluation metrics, with mean SSIM reaching 0.933 and mean PSNR reaching 35.44 dB. The results demonstrate the benefit of the multi-modal prior information fusion design of BL-INR: by integrating complementary information provided by multi-*b*-value DWIs and anatomical MRIs, the model can more accurately distinguish true signals from random noise, achieving a better balance between noise suppression and structure preservation.

### 3.6. Maximum frequency parameter and encoding strategy study

**Figure 11.**
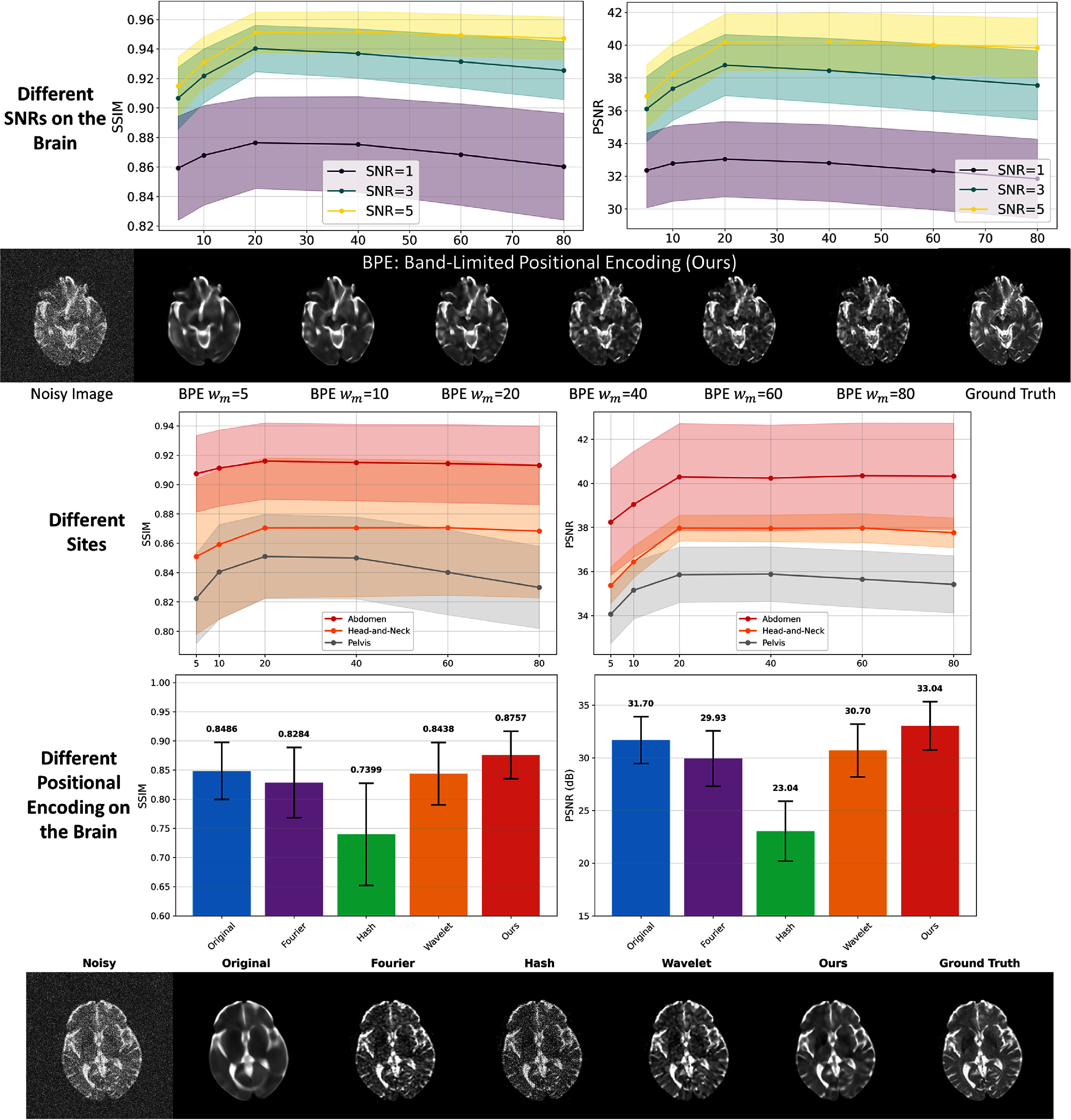
Parameter study and encoding strategy comparison for band-limited positional encoding. First row: Impact of $\omega_{\max}$ on SSIM and PSNR under different SNR levels (1, 3, 5) for brain data, with shaded regions indicating standard deviation. Second row: Visual effects at different $\omega_{\max}$ values (5, 10, 20, 40, 60, 80) for brain data at SNR = 1. Third row: Impact of $\omega_{\max}$ on SSIM and PSNR for Abdomen, head-and-neck, and Pelvis at SNR = 3. Fourth and fifth rows: Quantitative comparison and visual effects of different encoding strategies (Original coordinates, Fourier, Hash, Wavelet, Band-limited).

We systematically studied the impact of the maximum frequency parameter ($\omega_{\max}$) in BL positional encoding on model performance to optimize the frequency selection, validated its robustness under different SNR conditions, and conducted comprehensive comparisons with other advanced encoding strategies. Figure 11 presents the results of these comprehensive experiments.

The $\omega_{\max}$ parameter study reveals the relationship between frequency cutoff and denoising performance. The SSIM and PSNR curves exhibit an inverted U-shape, reaching performance peaks around $\omega_{\max} = 20$. When $\omega_{\max}$ is set too low (such as 5 or 10), the frequency range of positional encoding is overly restricted, resulting in insufficient high-frequency expression capability of the model. Correspondingly, the network cannot adequately capture mid-to-high frequency components containing important structural information in images, producing over-smoothed reconstruction results. Conversely, when $\omega_{\max}$ is set too high (such as 60 or 80), the frequency cutoff loses its effective noise suppression function, with large amounts of high-frequency noise components being learned by the INR model, reducing denoising effectiveness. Notably, although absolute performance varies across different SNR conditions and different anatomical regions (brain, head-and-neck, abdomen, pelvis), the optimal $\omega_{\max}$ value consistently remains around 20, with deviations from this value leading to performance degradation. This consistency across SNR levels and anatomical regions not only proves the robustness of our parameter selection but also indicates that adaptive adjustment based on different noise levels or anatomical regions may be less critical, while future investigations on a larger, more diverse dataset are warranted.

The encoding strategy comparison reveals the essential characteristics of different approaches. Original coordinate encoding suffers from its inherent low-frequency bias, leading to over-smoothing and resulting in lower SSIM = 0.8486 and PSNR = 31.70 dB. Fourier encoding, while enhancing high-frequency expression capability, indiscriminately introduces high-frequency noise. Hash encoding (Müller *et al* 2022) exhibits severe noise overfitting problems, likely because hash encoding greatly enhances INR’s high-frequency fitting capability, causing it to learn noise, resulting in an SSIM of only 0.7399 and a PSNR of only 23.04 dB. Wavelet encoding (Saragadam *et al* 2023), despite having multi-scale properties, still fits considerable noise. In contrast, our BL encoding, through optimized frequency cutoff achieved by the sinc function, effectively suppresses high-frequency noise while preserving important structural information, achieving optimal performance.

### 3.7. Patch size and registration robustness analysis

**Figure 12.**
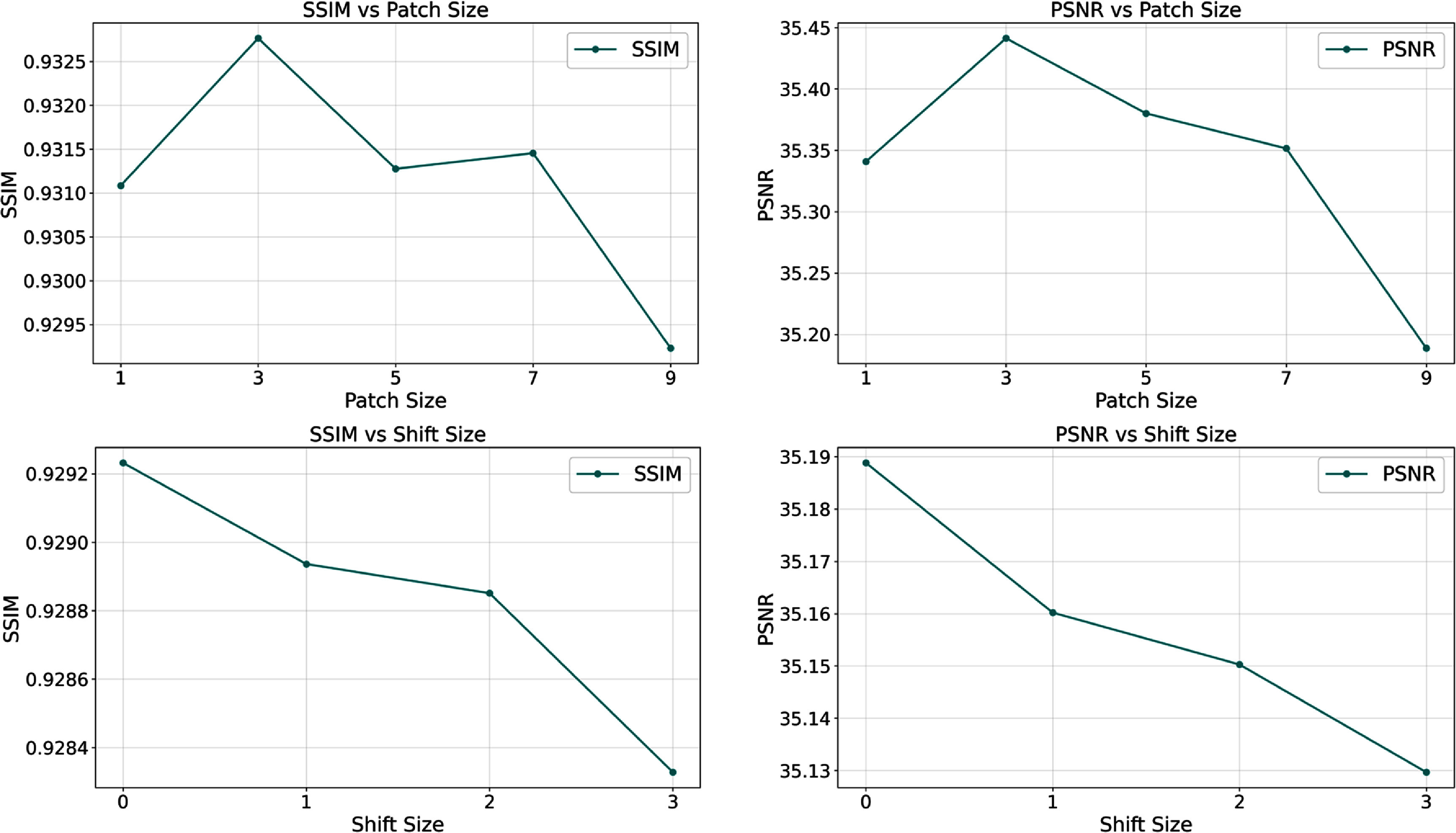
Impact of patch size and registration shifts on BL-INR denoising performance. The upper part shows SSIM and PSNR trend changes under different patch sizes (1, 3, 5, 7, 9), while the lower part presents the impact of different *Y*-axis direction shifts (0, 1, 2, 3 pixels) on performance when patch size is fixed at 9. Results indicate that patch size of 3 achieves optimal performance, while the model exhibits good robustness to small registration biases.

To comprehensively evaluate the clinical applicability of BL-INR, we systematically investigated the impact of patch size parameters on model performance and assessed the model’s robustness to potential registration biases between multi-modal images. This analysis is crucial for understanding BL-INR’s performance in real clinical scenarios, as clinical acquisitions often face challenges such as patient motion. Figure 12 presents quantitative analysis results of the impact of patch size selection and registration shifts on denoising performance. The patch size parameter study reveals a trade-off between spatial context and computational complexity. When the patch size is set to 1, the model only utilizes information from a single voxel for denoising, lacking sufficient spatial context information. As the patch size increases to 3, both SSIM and PSNR metrics reach their peak values, indicating that this size provides an optimal inclusion of neighborhood information. When the patch size further increases to 5, 7, and 9, the performance shows a declining trend. This performance degradation can be attributed to information redundancy introduced by excessively large receptive fields, increasing model learning complexity while potentially including information irrelevant to the target voxel, interfering with effective feature extraction and representation learning.

The registration robustness experiment simulates registration errors between multi-modal images in actual clinical practice by introducing progressive spatial shifts in the *Y*-axis direction. In the experimental setup, we fixed the patch size at 9 to provide a sufficient spatial buffer, then systematically evaluated the impact of shift amounts from 0 to 3 pixels on denoising performance. As shown in figure 12, although the denoising accuracy with patch size 9 is slightly inferior to the optimal patch size 3, the model still achieves effective denoising while demonstrating good robustness to registration biases. Specifically, as the shift increases from 0 to 3 pixels, SSIM only slightly decreases from 0.9293 to 0.9283 (a decline of 0.11%), and PSNR decreases from 35.19 dB to 35.13 dB (a decline of 0.17%). This minimal performance change confirms BL-INR’s inherent robustness to registration errors. This robustness stems from our patch-based processing strategy: as long as the prior information corresponding to the target voxel remains within the input patch’s receptive field, the model can effectively utilize multi-modal complementary information for denoising. Notably, this result embodies the trade-off in patch size selection between denoising accuracy and mis-registration tolerance: while larger patch sizes may reduce denoising accuracy under optimal conditions, they can significantly enhance the model’s tolerance to registration errors. This characteristic has important implications for clinical applications, as it reduces the strict requirement for perfect image registration, improving the method’s feasibility and practicality in actual clinical workflows.

## 4. Limitation and future work

Although this study has achieved promising results in DWI denoising, a key limitation remains. The patient-specific training strategy, while enabling individualized denoising, correspondingly increases computational costs. Each model training requires 10 minutes. Future research can include: developing fast fine-tuning strategies based on pre-trained models (Howard and Ruder 2018), exploring knowledge distillation techniques to construct lightweight models (Hinton *et al* 2015), or designing incremental learning frameworks to achieve rapid model adaptation (Kirkpatrick *et al* 2017). In addition, the core ideas of the BL-INR framework—frequency-selective neural representation and multi-modal prior information fusion—have broad application potential beyond DWI denoising. Future work can explore extending this framework to other medical image denoising or processing tasks.

## 5. Conclusion

By introducing BL positional encoding, the BL-INR method effectively suppresses high-frequency noise while maintaining INR’s powerful representation capabilities. Combined with multi-modal prior information fusion and self-supervised learning strategies, BL-INR allows ‘one-shot’ denoising without requiring paired training data. BL-INR outperformed existing methods on clinical DWI evaluations. Additionally, phantom measurements validated the accuracy of quantitative maps derived from DWIs denoised by BL-INR. BL-INR provides a practical solution to clinical DWI denoising and introduces a new technical paradigm of frequency-selective neural representation to the medical image processing field.

## Data Availability

The data cannot be made publicly available upon publication because they contain sensitive personal information. The data that support the findings of this study are available upon reasonable request from the authors.
